# Clinical Features and Gene Mutations of Lung Cancer Patients 30 Years of Age or Younger

**DOI:** 10.1371/journal.pone.0136659

**Published:** 2015-09-02

**Authors:** Yuehong Wang, Junjun Chen, Wei Ding, Bing Yan, Qiqi Gao, Jianying Zhou

**Affiliations:** 1 Department of Respiratory Medicine, The First Affiliated Hospital, College of Medicine, Zhejiang University, Hangzhou, China; 2 Department of Pathology, The First Affiliated Hospital, College of Medicine, Zhejiang University, Hangzhou, China; Zhongshan Hospital Fudan University, CHINA

## Abstract

**Purpose:**

Few studies examining the clinical features and gene mutations in lung cancer patients 30 years of age or younger have been published. A trend towards increasing morbidity has been noted in young patients; thus, an urgent need exists to explore this subgroup of patients.

**Methods:**

Patients aged ≤30 years with pathologically diagnosed lung cancer were retrospectively evaluated. We reviewed the clinical features, gene mutations and prognosis of each patient.

**Results:**

Forty-one patients were included in this study. The mean age was 26.4±3.5 years. Cough, tightness/dyspnea and chest pain were common symptoms, and 58.5% of patients presented with advanced stages of lung cancer. Adenocarcinoma was the predominant histologic type noted in these young patients. Masses and nodules were the dominant imaging features observed upon lung computed tomography (CT). Thoracic lymphadenopathy occurred very frequently in these patients. Five of 6 patients with echinoderm microtubule-associated protein-like 4 (EML4)-anaplastic lymphoma kinase (ALK) gene fusions presented solid masses with no ground-glass opacity (GGO) and thoracic multifocal lymphadenopathy. Six of 22 (27.2%) cases contained EML4-ALK gene fusions. In addition, 5 of 22 (22.7%) patients harbored epidermal growth factor receptor (EGFR) mutations, and 2 of 17 patients exhibited KRAS and ROS1 gene mutations. The median survival times were 44.2 months for patients with early stage disease and 8 months for patients with advanced NSCLC disease. The one-year and 5-year survival rates were 56.6% and 38.6%, respectively.

**Conclusions:**

Increased gene mutation frequencies are noted in these very young lung cancer patients. This finding indicates that the detection of gene mutations in these patients is important and will help to determine the appropriate targeted therapy.

## Introduction

Lung cancer is the leading cause of cancer-related deaths worldwide, with a 5-year survival rate of only 15% [1]. The annual lung cancer mortality rate in China is estimated to reach 1 million individuals by 2025 [2]. The majority of lung cancer cases occur in patients over 50 years old, most often between 60 and 80 years of age [3]. The incidence of lung cancer in young adults is reportedly relatively low. The incidence is approximately 1.2 to 6.2% in patients under 40 years of age [4,5], 5.3% in those under 45 [6,7], and 13.4% in those under 50 years [8]. However, some recent reports have indicated that the incidence of lung cancer in young patients is increasing worldwide [7,9–13]. Various studies have discussed the characteristics and prognosis of lung cancer in young patients <45 or <40 years old. Furthermore, lung cancer patients younger than 30 years old with poor prognoses have been reported [14–16]. Indeed, in recent years, we have also observed increasing numbers of very young lung cancer patients. Unfortunately, little research has been conducted regarding the clinical features, gene mutations and prognosis of this subgroup of patients due to the limited documented cases.

In recent years, targeted therapy has served as a major breakthrough in the management of non-small cell lung cancer (NSCLC) [17–19]. Patients with epidermal growth factor receptor (EGFR) mutations or echinoderm microtubule-associated protein-like 4 (EML4) and anaplastic lymphoma kinase (ALK) gene fusions exhibit increased progression-free survival (PFS) and overall survival (OS) after EGFR-tyrosine kinase inhibitor (TKI) (erlotinib or gefitinib) or crizotinib treatment [20–26]. However, data describing gene mutations in young patients are rare, including patients younger than 30 years of age. In this study, we retrospectively analyzed the clinical features, gene mutations and prognosis of 41 lung cancer patients aged 30 years or younger. We hope that this study will aid clinicians in improving awareness as well as diagnosis and treatment strategies in this patient population.

## Methods

### Patients and Data Collection

We retrospectively collected data from 41 patients 30 years of age or younger with pathologically diagnosed lung cancer between January 2008 and July 2014 at The First Affiliated Hospital, College of Medicine, Zhejiang University. The medical records of all patients included in this study were analyzed for the following data: (1) demographic data, including age and sex; (2) symptoms; (3) smoking status and family history; (4) tumor histology and disease stage; (5) radiological imaging; (6) gene abnormalities; and (7) overall survival. Disease was staged according to the seventh edition of the TNM (tumor, node, and metastasis) classification system. Smoking status was divided into two categories: non-smokers and smokers (including current and previous smokers). Family history was considered positive if any member of a patient’s family had a history of lung cancer. The Ethics Committee of The First Affiliated Hospital, College of Medicine, Zhejiang University approved this study. All patients provided written informed consent for the use of their clinical data and tumor tissues for research.

### Imaging Techniques and Analysis

Computed tomography (CT) was performed on 64-slice systems (Brilliance iCT and 64-channel systems), and intra-venous contrast was used in all patients. All CT scans were assessed for the presence of a mass (>30 mm in maximum dimension), nodule (≤30 mm in maximum dimension), ground-glass opacity (GGO) and lymphadenopathy. GGO was defined as a hazy increase in attenuation that did not obscure normal lung markings. Lymphadenopathy was defined as hilar or mediastinal lymph nodes >15 mm in the short-axis dimension.

### Gene Mutation Assessment

We examined EGFR mutations in exons 18 to 21 and KRAS mutations in codons 12 and 13 using a PCR-based pyrosequencing assay. Sequence analysis was performed using the PyroMark ID system (Qiagen, Hilden, Germany). Each case was identified as positive or negative by comparison with the wild-type sequence. EML4-ALK rearrangements were examined by fluorescence in situ hybridization (FISH) with a break-apart ALK probe (Vysis LSI Dual Color, Break Apart Rearrangement Probe; Abbott Molecular, Abbott Park, IL, USA). EML4-ALK rearrangements were classified as positive if greater than 15% of the tumor cells displayed split signals or isolated signals containing a kinase domain. ROS1 expression was assessed by immunohistochemistry.

### Statistical Analysis

The patients were followed until September 31, 2014 or the date of their death. OS was defined as the time from the date of diagnosis to the date of death or last visit. Survival curves were calculated according to the Kaplan-Meier method and compared using log-rank tests. Statistical analysis was performed using SPSS 18.0 software (SPSS, Chicago, IL).

## Results

### Patient Characteristics

A summary of the characteristics of 41 lung cancer patients is provided in Table 1. The age of the lung cancer patients assessed ranged from 17 to 30 years with a mean age of 26.4±3.5 years. In the entire cohort, 23 (56.1%) cases were males, and 18 (43.9%) cases were females. Only five patients (12.2%) reported a history of smoking, and only 1 patient had a family history of lung cancer. Cough was the most common initial presenting symptom, which occurred in 25 patients (61.0%), followed by chest tightness/dyspnea (24.4%), chest pain (21.9%) and bone/muscle pain (17.1%). Specifically, 12 patients (29.3%) were asymptomatic with abnormal chest radiological findings. Regarding disease stage, 22 (56.3%) NSCLC patients had advanced stage tumors (IIIb + IV) at presentation, and two small cell lung cancer (SCLC) patients exhibited extensive disease.

**Table 1:** The clinical characteristics of 41 patients.

Clinical characteristics	No.	(%)
**Mean age, years (range)**	26.4±3.5	
**Sex**		
Male	23	56.1
Female	18	43.9
**Smoking**		
Non-smoker	36	87.8
Current or ex-smoker	5	12.2
**Symptoms present**		
Cough	25	61.0
Chest tightness/dyspnea	10	24.4
Chest pain	9	22.0
Hemoptysis	5	12.2
Bone/muscle pain	7	17.1
Fever	6	14.6
No symptom	12	29.3
**Family history**	1	2.4
**NSCLC TNM stage**		
I	6	15.3
II	6	15.3
IIIa	5	12.8
IIIb	2	5.1
IV	20	51.2
**SCLC stage**		
Extensive	2	
Limited	0	

Furthermore, we analyzed the site of metastasis in 22 patients with advanced stage NSCLC, including four patients with ALK rearrangements, two with EGFR mutations, one with ROS1 mutations and 15 with unknown driver oncogenes. The majority of patients, 16 of 22 (72.7%), exhibited pulmonary nodules. Similar to a previous report [27], a large proportion of patients, 15 of 22 (68.2%), displayed metastasis to intrathoracic lymph nodes, whereas only 9 of 22 (40.9%) showed involvement of extrathoracic lymph nodes. Other common metastatic sites included bone (8/22, 36.4%), pleura (4/22, 18.2%) and brain (3/22, 13.6%). Additionally, we found an average of 2.33 metastatic sites among the 15 patients with unknown driver oncogenes and an average of 4 sites among the ALK rearrangement-positive patients; this difference was not statistically significant.

### Radiographic Findings

All 41 patients underwent contrast CT scans. The pulmonary abnormalities observed on the initial CT scans are summarized in Table 2. Masses were observed in 19 patients, with five patients exhibiting multiple nodules. Nodules were observed in 19 patients, with seven patients exhibiting solitary nodules and 12 patients exhibiting multi-scattered nodules; in addition, GGOs were observed in two patients. Most of the masses/nodules were ill-defined with irregular margins, and no cavitations were observed. Lymphadenopathy was observed in 27 patients. Other associated findings included segmental/lobar atelectasis (n = 4), pleural effusion (n = 6) and pericardial fluid build-up (n = 2).

**Table 2:** Radiological characteristics of 41 patients.

Major Radiographic Findings	No. of patients	%
Mass	19	46.3
Nodule	19	46.3
Multiple peripheral shadows	13	31.7
Lymphadenopathy	27	65.9
Segmental/lobar atelectasis	4	9.8
Pleural effusion	6	14.6
Pericardial fluid	2	4.9

**Fig 1:**
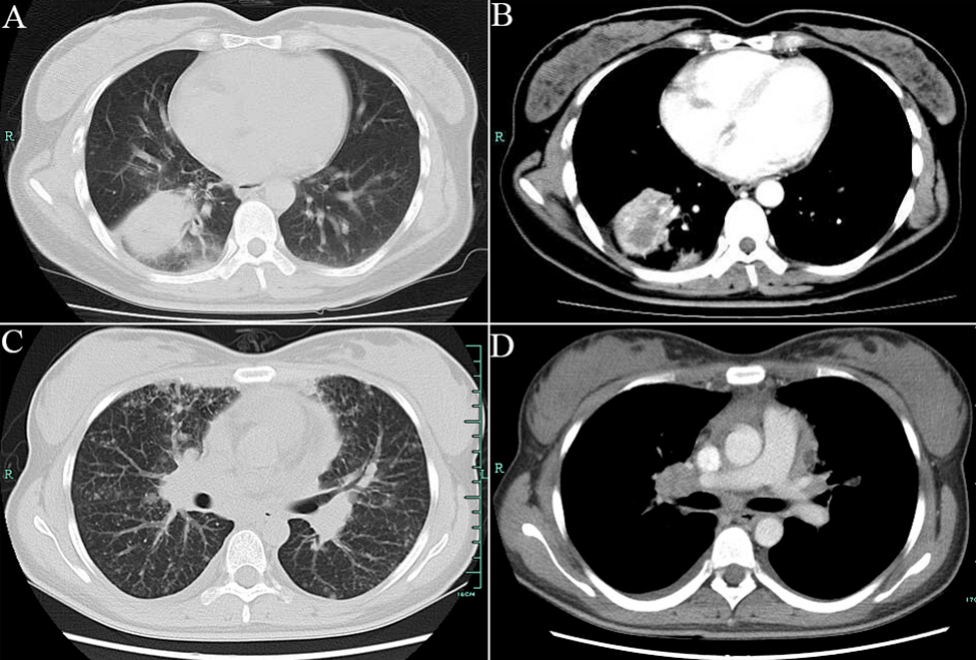
Thoracic CT findings of representative NSCLC patients with EML4-ALK gene fusions. A, B: mass with no GGO; C: multiple nodules; D: multifocal lymphadenopathy.

Five of 6 patients with EML4-ALK gene fusions presented solid masses with no GGO, and 1 of these patients exhibited multiple nodules. Five of 6 patients with EML4-ALK gene fusions also had thoracic multifocal lymphadenopathy (Fig 1).

Three of 5 patients with EGFR mutations showed a solitary nodule or mass, and two of these patients presented a mass or consolidation combined with multiple nodules and intrathoracic lymphadenopathy. All of the nodules were solid, and no GGO was observed in these patients.

### Histological Profiles

The predominant histologic type of lung cancer in these young patients was adenocarcinoma, accounting for 78.0% of cases (n = 32), followed by neuroendocrine carcinoma (n = 3, 7.3%), SCLC (n = 2, 4.9%) and undifferentiated NSCLC (n = 2, 4.9%). Specifically, only one patient exhibited squamous cell histology (Table 3).

**Table 3:** Pathological type of 41 patients.

	Male		Female	
Pathological type	N	%	N	%
Adenocarcinoma	18	43.9	14	34.1
Neuroendocrine carcinoma	1	2.4	2	4.8
SCLC	2	4.9	0	0.0
Undifferentiated NSCLC	1	2.4	1	2.4
Squamous cell carcinoma	0	0.0	1	2.4
Mucoepidermoid carcinoma	1	2.4	0	0.0

The histologic characteristics of the lung adenocarcinoma cases could be evaluated in 26 patients. Acinar predominant adenocarcinoma was the most common subtype (14/26, 53.8%), followed by solid predominant adenocarcinoma (8/26, 30.8%), lepidic predominant adenocarcinoma (1/26, 3.8%) and other subtypes (3/26, 11.5%). No papillary or micropapillary predominant adenocarcinomas were found in our study. Most patients with EGFR mutations presented as acinar predominant adenocarcinoma, while patients with EML4-ALK gene fusions tended to present as solid predominant adenocarcinoma. A lepidic growth pattern was found in two patients, both of whom harbored EGFR mutations. A signet ring cell feature was observed in three patients, 2 of whom harbored EML4-ALK gene fusions (S1 Table).

### Lung Adenocarcinoma Gene Mutations

Of the 32 adenocarcinoma patients studied, 22 had specimens available for gene mutation assessment (Table 4). A total of 22 cases were evaluated for EGFR mutations and EML4-ALK gene fusions. Five patients (3 males and 2 females) (5/22, 22.7%) harbored EGFR mutations, including two with L858R mutations and three with exon 19 deletions. Six of 22 cases (2 males and 4 females) (6/22, 27.2%) possessed EML4-ALK gene fusions. KRAS and ROS1 mutations were assessed in 17 patients. Two KRAS mutations and 2 ROS1 mutations were identified; both KRAS mutations were Gly12Asp.

**Table 4:** Gene alterations of 22 lung adenocarcinoma patients.

No.	Age	Sex	T	N	M	Stage	EGFR	ALK	KRAS	ROS1	Initial treatment
1	30	M	4	3	1a	IV	L858R	-	-	-	untreated
2	27	F	2	2	0	IIIa	L858R	-	-	-	radical surgery
3	30	M	4	3	1	IV	exon 19-del	-	-	-	gefitnib
4	27	M	2a	0	0	Ib	exon 19-del	-	-	-	radical surgery
5	27	F	1a	2	0	IIIa	exon 19-del	-	-	-	radical surgery
6	23	F	4	3	1b	IV	-	+	-	-	chemotherapy
7	29	F	4	3	1b	IV	-	+	-	-	chemotherapy
8	30	M	2a	2	0	IIIa	-	+	-	-	radical surgery
9	30	F	1b	2	0	IIIa	-	+	-	-	radical surgery
10	30	M	3	0	1a	IV	-	+	-	-	palliative surgery
11	23	F	4	3	1	IV	-	+	-	-	chemotherapy
12	30	M	2b	0	0	IIa	-	-	Gly12Asp	-	radical surgery
13	27	F	3	0	0	IIb	-	-	Gly12Asp	-	radical surgery
14	30	M	1a	0	0	Ia	-	-	-	+	radical surgery
15	27	M	1	3	1b	IV	-	-	-	+	crizotinib
16	30	F	4	2	1a	IV	-	-	-	-	chemotherapy
17	30	F	1a	0	0	Ia	-	-	-	-	radical surgery
18	26	M	2a	0	0	Ib	-	-	n.d.	n.d.	radical surgery
19	18	M	1a	1	0	IIa	-	-	n.d.	n.d.	radical surgery
20	25	M	4	3	1b	IV	-	-	n.d.	n.d.	untreated
21	28	F	3	0	0	IIb	-	-	n.d.	n.d.	radical surgery
22	30	M	3	3	1b	IV	-	-	n.d.	n.d.	chemotherapy

n.d.: not detected

### Treatment and Overall Survival Analysis

For the initial treatment of the NSCLC patients, 18 patients received surgical therapy, 14 patients received chemotherapy, 2 patients received targeted agents (gefitinib and crizotinib for each) and 5 patients quit therapy (Table 5). For second line therapy, 3 patients with ALK rearrangements received crizotinib. Both SCLC patients initially received chemotherapy.

**Table 5:** The treatment of 39 NSCLC patients.

Treatment	No.
Surgery	18
no adjuvant therapies	5
surgery+adjuvant therapies	5
unclear adjuvant therapies	7
Chemotherapy	14
Targeted therapy (2nd line)	3
Targeted therapy (1st line)	2
Untreated	5

**Fig 2:**
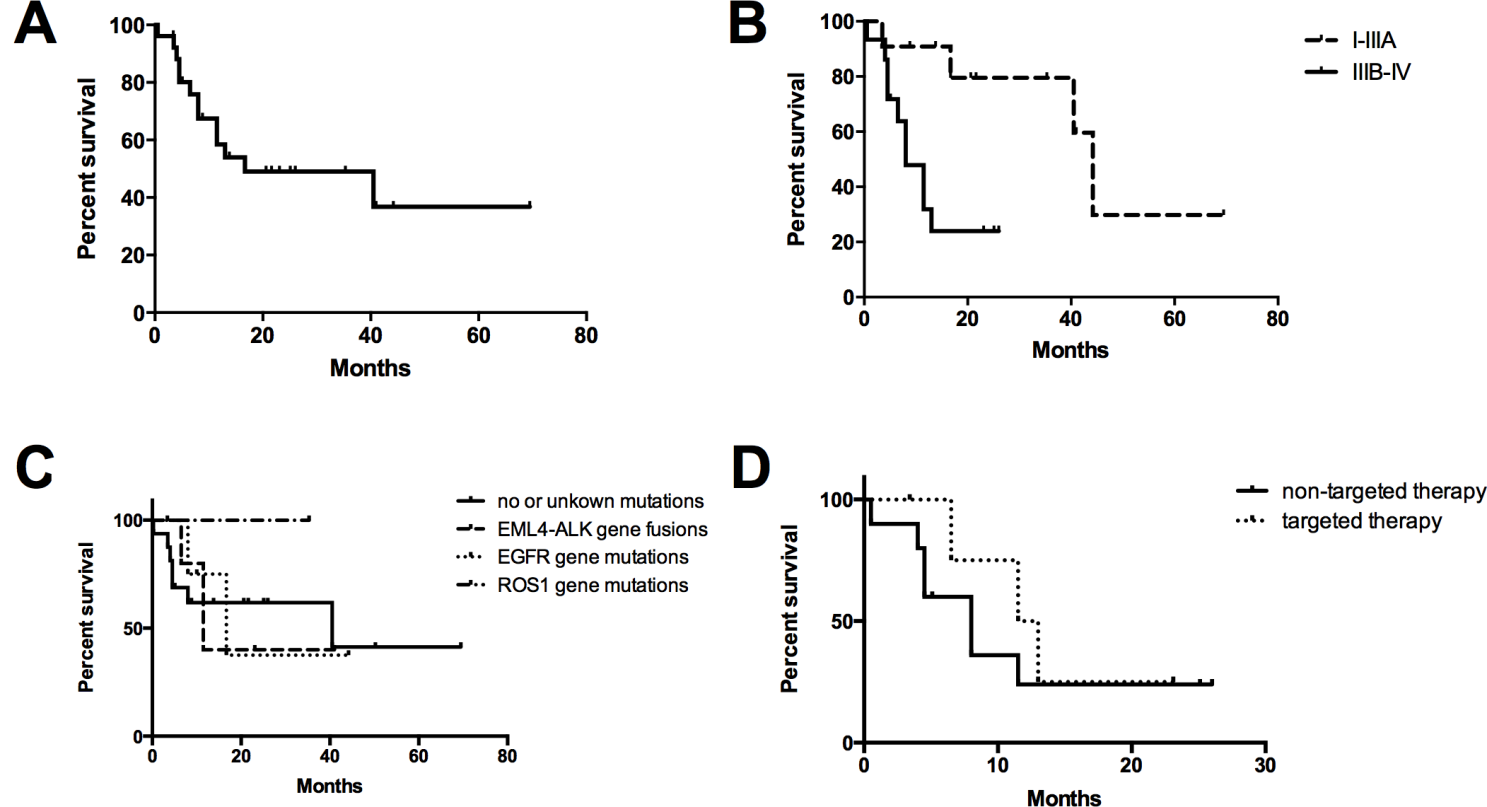
Survival curves for lung cancer patients are presented. A: Overall survival curves for 25 NSCLC patients; B: Survival by stage; C: Survival by oncogenic drivers; D: Survival by targeted therapy.

Long-term follow-up data were available for 25 NSCLC patients (Fig 2). The follow-up time ranged from 1 to 70 months (median time of 14 months). Twelve deaths occurred during the follow-up period. The median survival time was 44.2 months for patients with early stage disease and 8 months for those with advanced disease. The one-year and 5-year survival rates were 56.6% and 38.6%, respectively. Fifteen NSCLC patients with advanced disease were available for survival analysis, in which 5 patients received targeted agents (1 with gefitinib and 4 with crizotinib). The median survival time was 12.25 months and 8 months for the targeted therapy cohort and non-targeted therapy cohort, respectively. However, no statistical significance was observed.

## Discussion

Here, we report the clinical features and gene mutations of lung cancer patients 30 years of age or younger. We found that no specific clinical symptoms were ascribed to these patients. Cough was the most common initial clinical presentation, followed by chest tightness/dyspnea and chest pain, which is similar to observations reported in previous studies [28,29]. The manifestations of lung cancer on chest CT in younger patients were similar to those observed in older patients. According to our study, masses or nodules were the most common CT manifestations. Multiple peripheral shadows, which may be easily confused with inflammatory lung disease, were also frequently noted. In particular, thoracic lymphadenopathy frequently occurred in these patients, and this finding may be related to thoracic lymph node metastasis. Recently, Hsu and Togashi [30,31] reported that lung adenocarcinomas in patients with EGFR mutations tend to have an invasive solid pattern at early stages and diffuse and random pulmonary metastases at advanced stages. This trend was also observed in our patients. Furthermore, few studies have described the imaging findings related to EML4-ALK gene fusions in lung cancer. In the present study, 5 of 6 patients with EML4-ALK mutations exhibited solid masses with no GGO lesions, and 1 of 6 patients harbored multiple nodules. Five of 6 ALK-positive patients had thoracic lymphadenopathy. These features indicate that most ALK-positive tumors exhibit a solid growth pattern without GGO and have a tendency to infiltrate into localized lymph nodes. Furthermore, compared to patients with EGFR mutations, lymphadenopathy was more common and remarkable in patients with EML4-ALK gene fusions in our study. Therefore, a solid mass and thoracic lymphadenopathy may be the most common imaging features of ALK-positive patients, as indicated by Fukui and Park et al. [32,33]. Given the nonspecific clinical presentations, these very young patients are typically misdiagnosed with pneumonia or pulmonary tuberculosis. Moreover, disease in a younger patient is less likely to be considered cancer when symptoms occur because physicians typically consider cancer at the end of a differential diagnosis. As a result, the correct diagnosis of lung cancer in patients younger than 30 years of age is often delayed. The median interval time from symptom onset to diagnosis was greater than 30 days in our series.

In our study, adenocarcinoma was the leading histologic type, accounting for greater than 70% of all cases. In addition, squamous cell carcinoma was rarely observed. Previous studies [14–16,28] have also demonstrated that younger patients exhibit a greater incidence of adenocarcinoma (33–82.6%) and a low proportion of squamous cell carcinoma lesions (6.3–22%). Although the reason for the high percentage of adenocarcinoma in younger patients is unclear, we believe that smoking is one factor involved in this phenomenon. Given that the development of lung cancer may occur over decades after beginning to smoke, an increased number of adenocarcinoma lesions and fewer smoking-related squamous cell carcinoma lesions may be expected in young patients. Subramanian et al. [5] and McDuffie et al. [34] reported that lung cancer patients with no history of smoking appear to develop lung cancer at earlier ages compared with lung cancer patients with a history of smoking. Genetic and environmental factors have been suggested to play important roles in young patients with lung cancer. Furthermore, in studies by Y. Pan and Kim [35,36], the most common histologic patterns observed were acinar and solid predominant adenocarcinoma. In addition, ALK-rearranged tumors more frequently showed a solid predominant pattern and signet ring cells, which is similar to observations reported in previous studies [36,37].

Interestingly, our study demonstrated that EGFR and EML4-ALK mutations were observed in 50% (11/22) of the lung adenocarcinoma patients. EGFR mutation frequencies in lung adenocarcinoma reported in various studies have exhibited significant variations due to ethnicity, sex and smoking status. In unselected lung adenocarcinoma cases, EGFR mutations are present in ~15% of Caucasian cases and 30 to 50% of East Asian cases [17,18,38]. Moreover, ALK rearrangement is observed in 3 to 5% of unselected NSCLC cases [19,39]. VandenBussche et al. [40] reported that the frequency of EGFR mutations and ALK translocations is increased among Caucasian patients aged <50 years. Interestingly, studies have also demonstrated that EML4-ALK and ROS1 gene rearrangement mutations are significantly more common in young Asian patients [41,42]. Our study revealed that EML4-ALK rearrangement was the most common mutation (27.2% of cases), followed by EGFR mutations (25% of patients). Compared with overall gene alterations in Asian lung adenocarcinoma patients, EML4-ALK alterations in our study were relatively increased, and EGFR mutations were relatively reduced. In addition, EML4-ALK alterations predominantly occurred in female patients [41,42]. Ye T and his colleagues [43] reported the molecular characteristics of 36 resected lung adenocarcinomas from young patients under 40 years old. The mean age was 34.53±4.63, and only one patient had advanced stage disease. Their research showed that EGFR mutations occurred in greater than 50% of patients, and ALK rearrangements occurred in only 5.6% of patients. Several reasons may exist for the discrepancies between their study and our study: (1) This results may be influenced by the age of the population studied. The mean age in our study was 26.4±3.5, compared to 34.53±4.63 in the study by Ye T et al. Meanwhile, Nagashima [41] and Sholl [42] demonstrated that EML4-ALK and ROS1 gene rearrangement mutations are significantly more common in young patients. (2) A higher percentage of advanced stage disease was observed in our study (greater than 50% of all patients). (3) Of note, the study size may have been an important influential factor in both studies. ROS1 rearrangement is observed in 1 to 2% of unselected NSCLC cases [37,44,45]. KRAS mutations are present in 25–40% of adenocarcinoma patients, and these mutations are rarely observed in never-smokers [46]. In this study, 2 of 17 patients harbored KRAS and ROS1 mutations. ROS1 gene mutations occurred more frequently in our study compared with the overall lung cancer patient population. The high frequency of gene mutations in our study indicates that the detection of gene mutations in these very young patients is important and will help to identify the appropriate targeted drug therapy, such as EGFR-TKI and crizotinib.

In this study, long-term follow-up data were available for 25 patients. The median survival time was 8 and 44.2 months in advanced disease stages and early disease stages, respectively. According to a previous study that included 20 patients 30 years of age or younger [16] with either stage III or IV disease, the median survival was only 5.5 months, and no 5-year survivors were noted. The poor prognosis was partially related to the lack of targeted therapies at that time. The 1-year and 5-year survival rates in our study were 56.6% and 38.6%, respectively. Zhang et al. [7] have reported 1-year and 5-year survival rates of 49.87% and 23.12%, respectively, in patients younger than 45 years. The results in our study should be interpreted carefully given the small number of patients studied.

## Conclusions

Higher gene mutation frequencies are found in these very young lung cancer patients. EML4-ALK gene fusion was the most common mutation. This study indicates that it is very important to detect the gene mutations in young patients, and it will help to determine the appropriate therapy. As far as we know, no study has described the gene mutation characteristics of lung cancer among patients 30 years of age or younger, and our study has filled this gap. However, this study has several limitations. First, the study utilized a retrospective design. Second, the sample size was small. Thirdly, only a small number of patients received the targeted therapy. Further prospective studies are needed to identify gene alterations and therapeutic strategies in this subgroup of patients.

## Supporting Information

S1 Table Pathologic characteristics of 26 lung adenocarcinoma patients. (XLSX)
